# P-2054. Effectiveness of a Single COVID-19 mRNA Vaccine Dose in Individuals Previously Infected with SARS-CoV-2: A Systematic Review

**DOI:** 10.1093/ofid/ofae631.2210

**Published:** 2025-01-29

**Authors:** Hannah R Volkman, Jennifer L Nguyen, Mustapha M Mustapha, Jingyan Yang, Luis Jodar, John M McLaughlin

**Affiliations:** Pfizer, New York, New York; Pfizer Inc., New York, New York; Pfizer, New York, New York; Pfizer and Columbia University Institute for Social and Economic Research and Policy, New York, New York; Pfizer Vaccines, Collegeville, Pennsylvania; Pfizer, New York, New York

## Abstract

**Background:**

Based on the high levels of population immunity to SARS-CoV-2 from prior infection in many countries, in fall 2023 the US Food and Drug Administration, European Medicines Agency, and World Health Organization authorized/recommended a single mRNA XBB.1.5-adapted vaccine dose for unvaccinated individuals aged ≥ 5y.

**Methods:**

This systematic literature review evaluated the vaccine effectiveness (VE) of a single COVID-19 mRNA dose (BNT162b2 or mRNA-1273) in individuals previously infected with SARS-CoV-2 compared to those with differing histories of prior infection and vaccination (PROSPERO ID: CRD42023453257). We searched MEDLINE and Embase for VE studies published from January 1, 2021–October 4, 2023. Data were synthesized following Synthesis Without Meta-Analysis guidelines. Bias was assessed using the Newcastle-Ottawa Scale. VE estimates were reported as a range of point estimates per clinical endpoint and meta-analyses were not performed due to heterogeneity.

**Results:**

Of 1,661 initial results, 18 studies were eligible for inclusion. Among those previously infected, a single mRNA dose (compared to being unvaccinated) increased protection by 8–71% against infection (related to Omicron BA.1, BA.4/5, or XBB), 39–67% against symptomatic infection (BA.1, BA.2, or BA.4/5), and 25–60% against hospitalization or hospitalization or death (BA.1). The protection provided by a single dose among those previously infected was comparable to the VE provided by two doses, and was higher than VE following two doses without prior infection. No studies were identified that reported single-dose VE for adapted vaccines (BA.1 or BA.4/5 bivalent or XBB.1.5-adapted formulations), immunocompromised populations, or children aged < 5y.

**Conclusion:**

Our results suggest that a single dose of COVID-19 vaccine provides similar protection to that conferred by a two-dose series for immunocompetent individuals aged ≥ 5y in the current setting of high pre-existing SARS-CoV-2 immunity. These findings support current recommendations for one dose of COVID-19 vaccines regardless of vaccination status to be given in advance of the viral respiratory season with considerations for additional doses for certain special populations including young children, older adults, and the immunocompromised.

**Disclosures:**

Hannah R. Volkman, PhD, MPH, Pfizer Inc: Employment|Pfizer Inc: Stocks/Bonds (Public Company) Jennifer L Nguyen, ScD, MPH, Pfizer Inc: Employment|Pfizer Inc: Stocks/Bonds (Public Company) Mustapha M. Mustapha, MD, PhD, MPH, Pfizer Inc: Employment|Pfizer Inc: Stocks/Bonds (Public Company) Jingyan Yang, MHS, DrPH, Pfizer Inc: Employment|Pfizer Inc: Stocks/Bonds (Public Company) Luis Jodar, PhD, Pfizer Inc: Employment|Pfizer Inc: Stocks/Bonds (Public Company) John M. McLaughlin, PhD, Pfizer: Employee|Pfizer: Stocks/Bonds (Public Company)

